# Hypercapnia induces IL-1β overproduction via activation of NLRP3 inflammasome: implication in cognitive impairment in hypoxemic adult rats

**DOI:** 10.1186/s12974-017-1051-y

**Published:** 2018-01-05

**Authors:** Hong-Guang Ding, Yi-Yu Deng, Ren-qiang Yang, Qiao-Sheng Wang, Wen-Qiang Jiang, Yong-Li Han, Lin-Qiang Huang, Miao-Yun Wen, Wen-Hong Zhong, Xu-Sheng Li, Fan Yang, Hong-Ke Zeng

**Affiliations:** 1 Southern Medical University, 1838 North Guangzhou Avenue, Guangzhou, 510515 China; 2 Department of Emergency and Critical Care Medicine, Guangdong General Hospital and Guangdong Academy of Medical Sciences, 106 ZhongshanEr Road, Guangzhou, 510080 China; 3 Department of Emergency, Dongguan Third People’s Hospital, Dongguan, Guangdong China

**Keywords:** ARDS, Hypercapnia, Cognitive impairment, NLRP3 inflammasome, IL-1β

## Abstract

**Background:**

Cognitive impairment is one of common complications of acute respiratory distress syndrome (ARDS). Increasing evidence suggests that interleukin-1 beta (IL-1β) plays a role in inducing neuronal apoptosis in cognitive dysfunction. The lung protective ventilatory strategies, which serve to reduce pulmonary morbidity for ARDS patients, almost always lead to hypercapnia. Some studies have reported that hypercapnia contributes to the risk of cognitive impairment and IL-1β secretion outside the central nervous system (CNS). However, the underlying mechanism of hypercapnia aggravating cognitive impairment under hypoxia has remained uncertain. This study was aimed to explore whether hypercapnia would partake in increasing IL-1β secretion via activating the NLRP3 (NLR family, pyrin domain-containing 3) inflammasome in the hypoxic CNS and in aggravating cognitive impairment.

**Methods:**

The Sprague-Dawley (SD) rats that underwent hypercapnia/hypoxemia were used for assessment of NLRP3, caspase-1, IL-1β, Bcl-2, Bax, and caspase-3 expression by Western blotting or double immunofluorescence, and the model was also used for Morris water maze test. In addition, Z-YVAD-FMK, a caspase-1 inhibitor, was used to treat BV-2 microglia to determine whether activation of NLRP3 inflammasome was required for the enhancing effect of hypercapnia on expressing IL-1β by Western blotting or double immunofluorescence. The interaction effects were analyzed by factorial ANOVA. Simple effects analyses were performed when an interaction was observed.

**Results:**

There were interaction effects on cognitive impairment, apoptosis of hippocampal neurons, activation of NLRP3 inflammasome, and upregulation of IL-1β between hypercapnia treatment and hypoxia treatment. Hypercapnia + hypoxia treatment caused more serious damage to the learning and memory of rats than those subjected to hypoxia treatment alone. Expression levels of Bcl-2 were reduced, while that of Bax and caspase-3 were increased by hypercapnia in hypoxic hippocampus. Hypercapnia markedly increased the expression of NLRP3, caspase-1, and IL-1β in hypoxia-activated microglia both in vivo and in vitro. Pharmacological inhibition of NLRP3 inflammasome activation and release of IL-1β might ameliorate apoptosis of neurons.

**Conclusions:**

The present results suggest that hypercapnia-induced IL-1β overproduction via activating the NLRP3 inflammasome by hypoxia-activated microglia may augment neuroinflammation, increase neuronal cell death, and contribute to the pathogenesis of cognitive impairments.

## Background

Permissive hypercapnia is a ventilation strategy to allow for an unphysiologically high-partial pressure of carbon dioxide (PaCO_2_) included by reducing tidal volume, which serves to reduce pulmonary morbidity for acute respiratory distress syndrome (ARDS) patients [1]. Current guidelines recommend the concept of low tidal volume ventilation and permissive hypercapnia for patients with ARDS [2]. The benefits gained from the ventilation strategy are apparent; on the other hand, hypercapnia may present a risk to CNS. It has been reported in various studies that hypercapnia contributes to the risk of cognitive impairment [3, 4]; indeed, half of all ARDS survivors develop cognitive impairments [5]. The incidence rate of cognitive impairment in ARDS survivors is over 70% at hospital discharge, over 46% at 1 year and over 20% at 2 years [6]. The mechanism of cognitive impairments in ARDS is still unclear. Some studies have found that a longer duration of hypoxemia was associated with cognitive impairment in ARDS survivors [7, 8]. It remains to be ascertained whether hypercapnia would aggravate cognitive impairment in ARDS patients with persistent hypoxemia.

It is well recognized that when activated by different external stimuli, microglia secrete a large number of pro-inflammatory cytokines (i.e., IL-1β), and exposure of brain to hypoxemia represents one such stimulus, which is typical clinical presentation of ARDS. Previous studies have demonstrated that permissive hypercapnia may contribute to IL-1β secretion. In the lung of endotoxemic rats, IL-1β expression was significantly upregulated by hypercapnia treatment [9]. This suggests that hypercapnia may enhance the release of IL-1β in the hypoxic CNS. The expression levels of IL-1β [10] and its receptor [11] are comparatively higher in the hippocampus and are tightly linked to the deficits in hippocampus-dependent memory [12]. In this connection, intra-ventricular infusion of the IL-1 receptor antagonist can block the deficits [13]. Increasing evidence suggests that IL-1β plays a pivotal role in inducing neuronal apoptosis in many neurodegenerative diseases [14]. It is well documented that Bcl-2 protein, as a gatekeeper of the mitochondrial pathway of apoptosis, has significant anti-apoptosis effect. However, Bax exhibits pro-apoptotic action. The ratio of Bax to Bcl-2 determines the downstream activation of caspase-3 [15]. Under pathological conditions, a large number of cell death may be caused by over-activation of apoptosis. In the CNS, over-activation of apoptosis can result in the death of a mass of neurons in the hippocampus CA1 regions and in cognitive impairment [16].

In the brain, pro-IL-1β, as an inactive form of IL-1β, is primarily produced by microglia in response to an inflammatory stimulus [17]. To exert its functions, pro-IL-1β requires cleavage to an active form by caspase-1, which is regulated by NLRP3 inflammasome [18, 19]. The core structure of the NLRP3 inflammasome is formed by three proteins: NLRP3, pro-caspase-1, and the adaptor protein ASC (apoptosis-associated speck-like protein containing a CARD). Extracellular ATP, urate, potassium efflux, or production of reactive oxygen species (ROS) can trigger the NLRP3 inflammasome, which would result in activation of caspase-1 and processing of pro-IL-1β to IL-1β [20, 21]. It has remained to be explored whether hypercapnia has the effect of activating the NLRP3 inflammasome specifically in the activated microglia in production of IL-1β.

In the present study, we hypothesized that hypercapnia may aggravate the cognitive impairment in adult male Sprague-Dawley (SD) rats with hypoxemia. It was surmised that hypercapnia may exert its effect through increasing IL-1β secretion, and via activating the NLRP3 inflammasome, it can cause excessive apoptosis of hippocampal neurons.

## Methods

### Animals and experimental groups

Adult male Sprague-Dawley (SD) rats weighing 250–300 g were provided by Institute of Laboratory Animal Science of Jinan University. The rats (*n* = 128) were randomly divided into four groups (*n* = 32 rats each): sham-operated group (abbreviated S group), exposed to the air; hypercapnia group, exposed to CO_2_ concentrations of 5% of the gas mixture to maintain pH at 7.2–7.25; hypoxemia group, exposed to O_2_ concentrations of 16% to maintain partial pressure of oxygen (PaO_2_) at around 60 mmHg. In the hypercapnia + hypoxemia group (abbreviated HH group), a gas mixture of 5% CO_2_ containing 16% O_2_ was used to maintain pH at 7.2–7.25 and PO_2_ at around 60 mmHg.

### Rat model of hypercapnia/hypoxemia

Before the experiments, all rats were fasted overnight but allowed free access to water. The rats were anesthetized with an intraperitoneal injection of 30 mg/kg pentobarbital sodium (Merck, Darmstadt, Germany; cat. no.1063180500) and were mechanically ventilated for 3 h using a small-animal ventilator (SAR-1000, CWE, Ardmore, PA, USA). The tidal volume was set at 9 ml/kg body weight, the respiratory rate was 45 breaths/min, and inspiratory to expiratory ratio was 1:1 [22]. Mechanical ventilation was performed using a gas tank containing either room air (S group), a gas mixture containing 5% CO_2_, 21% O_2_, 74% N_2_ (Hypercapnia group), 16% O_2_, 84% N_2_ (Hypoxemia group), or 5% CO_2_, 16% O_2_, and 79% N_2_ (HH group). Two gas monitors (P110, P120, Biospherix, Lacona, NY, USA) were used to monitor the concentrations of CO_2_ and O_2_ in the respiratory circuit.

The left femoral artery was cannulated with a self-made infusion tube (a PE 10 cannula and an indwelling needle connected with parafilm) to collect arterial blood samples. A Blood Gas/Electrolyte Analyzer (Model 5700, IL, San Diego, CA, USA) was used to measure PO_2_, PCO_2_, and pH of the arterial blood samples. The caudal vein was cannulated to enable an infusion of 0.9% saline for maintenance fluid. All surgical incisions were infiltrated with 0.25% lidocaine. The rats used for the following experiments (Morris water maze test, immunofluorescence staining, and Western blotting analysis) were mechanically ventilated without any kind of invasive manipulation. The rectal temperature was continuously measured and maintained at 37 °C using a heating lamp.

### Morris water maze test

Morris water maze (MWM) test was conducted to assess hippocampus-dependent spatial learning and memory functions in rodents [23]. The apparatus consisted of a 200 cm in diameter circular pool and a camera, which was placed above the pool and connected to a computer to track the behavior of rats. The pool was filled with warm water (25 ± 1 °C) and artificially divided into four equal quadrants. An escape platform, 9 cm in diameter, was permanently placed in one quadrant and 1 cm under the water level. Twenty-four hours after the treatment, each rat was allowed to swim in the water for 120 s to familiarize with the environment and task. At 48 and 72 h after treatment, rats were tested in the MWM task. Time taken by rats to reach the platform was recorded as latency time. If the rat failed to find the platform in 120 s, latency time was recorded as 120 s. The rat was manually guided to the platform and was allowed to stay on it for 60 s.

### Microglial culture and treatment

BV-2 microglial cells (CHI Scientific, Wuxi, China; cat. no. 7-1502) were cultured in Dulbecco’s Modified Eagle Medium (DMEM) high glucose (Gibco/Thermo Fisher, Grand Island, NY, USA; cat. no. 8117046) supplemented with 10% fetal bovine serum (FBS) (Capricorn Scientific GmbH, Ebsdorfergrund, Germany; cat. no. FBS-52A) at 37 °C in a humidified incubator with 5% CO_2_/95% air. The cells were divided into five groups: control group, exposed to 20% O_2_ + 5% CO_2_; high concentration of carbon dioxide group (HC group), exposed to 20% O_2_ + 15% CO_2_; hypoxia group, exposed to 0.2% O_2_ + 5% CO_2_; hypoxia + HC group, exposed to 0.2% O_2_ + 15% CO_2_; hypoxia + HC + Z-YVAD-FMK group, treated with Z-YVAD-FMK (10 μM) [24] (ApexBio, Boston, MA, USA; cat. no. A8955) for 30 min before being exposed to 0.2% O_2_ + 15% CO_2_. The treated cells from different groups were incubated in an air-tight chamber, in which the O_2_ and CO_2_ concentrations were controlled by a Carbon Dioxide and Oxygen Controller (ProOx C21, Biospherix, Lacona, NY, USA). A Blood Gas/Electrolyte Analyzer (Model 5700, IL, San Diego, CA, USA) was used to measure PO_2_, PCO_2_, and pH of supernatants.

### Preparation of BV-2 conditioned medium

Microglia-conditioned medium was prepared according to the procedure reported previously [25]. BV-2 microglial cells were cultured in 75 cm^2^ culture flasks with DMEM/F12 medium (Gibco/Thermo Fisher, Grand Island, NY, USA; cat. no. C11330500BT) supplemented with 10% FBS. The cells were washed with PBS to eliminate sera and incubated with DMEM/F12 medium without FBS. Five different kinds of conditioned medium were prepared: BV-2 conditioned medium (CM), BV-2 cells were exposed to 20% O_2_ + 5% CO_2_ for 24 h; BV-2 conditioned medium + high concentration of carbon dioxide (CM + HC): BV-2 cells were exposed to 20% O_2_ + 15% CO_2_ for 24 h; BV-2 conditioned medium + hypoxia (CM + hypoxia): BV-2 cells were exposed to 0.2% O_2_ + 5% CO_2_ for 24 h; BV-2 conditioned medium + hypoxia + high concentration of carbon dioxide (CM + hypoxia + HC): BV-2 cells were exposed to 0.2% O_2_ + 15% CO_2_ for 24 h; BV-2 conditioned medium with Z-YVAD-FMK pretreatment + hypoxia + high concentration of carbon dioxide (CM + hypoxia + HC + Z): BV-2 cells were treated with Z-YVAD-FMK (10 μM) for 30 min, the medium was then discarded and the microglial cultures were washed with PBS twice. Following this, 10 ml DMEM/F12 medium without FBS was added. The cells were exposed to 0.2% O_2_ + 15% CO_2_ for 24 h. The conditioned medium was filtered through 0.22 μm syringe filters and used immediately.

### Primary cultures of neurons and treatment with BV-2 conditioned medium

Primary neuronal cell cultures were prepared as described previously [26]. Briefly, the hippocampus was isolated from the brain of newborn SD rats (1 day old). The hippocampus was minced into tiny particles and digested with 0.125% trypsin for 10 min at 37 °C, then neutralized with fetal bovine serum (FBS), and finally centrifuged at 1100 rpm for 5 min. The cells were resuspended in DMEM/F12 medium containing 10% FBS and plated into poly-L-lysine (Sigma, St. Louis, MO, USA; cat. no. P1399) coated flasks. Cells were incubated at 37 °C in a humidified incubator with 5% CO_2_/95% air for 6 h. Then, the medium was changed and cells were incubated in neurobasal medium (Gibco/Thermo Fisher, Grand Island, NY, USA; cat. no. 121103049) containing 2% B27 (Gibco/Thermo Fisher, Grand Island, NY, USA; cat. no. 17504044) and 1% glutamine (Sigma, St. Louis, MO, USA; cat. no. G3126); half of the medium was replaced with neurobasal containing 2% B27 without glutamine 3 days later. Thereafter, they were incubated for 3 h in the following conditions: BV-2 conditioned medium (CM group), BV-2 conditioned medium + high concentration of carbon dioxide (CM + HC group), BV-2 conditioned medium + hypoxia (CM + hypoxia group), BV-2 conditioned medium + hypoxia + high concentration of carbon dioxide (CM + hypoxia + HC group), BV-2 conditioned medium with Z-YVAD-FMK pretreatment + hypoxia + high concentration of carbon dioxide (CM + hypoxia + HC + Z group). The purity of neurons was assessed by immunocytochemical staining using MAP-2 (a marker of neurons) (Abcam, Cambridge, MA, USA; cat. no. ab32454) and DAPI, a nuclear marker of all cells. The purity of primary neuron cultures was above 95% in this study.

### Western blotting analysis

Total proteins from hippocampus tissue, BV-2 cells and primary neuronal cells (*n* = 4 for each group) were extracted using a Total Protein Extraction Kit (BestBio, Shanghai, China; cat. no. BB-3101). Protein concentration was measured using a BCA Protein Assay kit (Bioworld Technology, St. Louis Park, MN, USA; cat. no. BD0028). Briefly, the protein samples were heated at 95 °C for 5 min. Equal amounts of protein was separated in a 12% SDS polyacrylamide gel and blotted onto PVDF membranes. Membranes were blocked with 5% non-fat milk for 1 h at room temperature and then incubated with primary antibodies overnight at 4 °C with light shaking. The primary antibodies used were as follows: IL-1β (applied to tissue, 1:1000, Abcam, Cambridge, MA, USA; cat. no. ab9787), IL-1β (applied to cells, 1:1000, Chemicon International, Temecula, CA, USA; cat. no. AB1832P), caspase-1 (1:1000, Abcam, Cambridge, MA, USA; cat. no. ab1872), NLRP3 (1:1000, Abcam, Cambridge, MA, USA; cat. no. ab214185), Bax (1:1000, Cell Signaling Technology, Danvers, MA, USA; cat. no. 14796), Bcl-2 (1:1000, Abcam, Cambridge, MA, USA; cat. no. ab194583), caspase-3 (1:1000, Cell Signaling Technology, Danvers, MA, USA; cat. no. 9664S). On the following day, the membranes were incubated with the horseradish peroxidase (HRP)-conjugated secondary antibodies for 2 h at 4 °C. The secondary antibody was goat anti-rabbit IgG-HRP (1:3000, Cell Signaling Technology; cat. no. 7074S). Protein was visualized with enhanced chemiluminescence using a chemiluminescence kit (Millipore, Billerica, MA, USA; cat. no. WBKLS0500), and images were generated by an imaging densitometer (ImageQuant LAS 500, GE Healthcare Bio-Sciences AB, Uppsala, Sweden). The relative density was quantified by FluorChem 8900 software (version 4.0.1, Alpha Innotech Corporation, San Leandro, CA, USA). In addition, GAPDH was used as the control.

### Immunofluorescence staining

Double immunofluorescence staining was carried out to detect IL-1β expression in microglia and caspase-3 expression in neurons in hippocampus tissue. After 3 h of treatment, four rats from each group were randomly selected. The rats were deeply anesthetized with pentobarbital sodium and transcardially perfused with ice-cold 0.9% saline rapidly followed by 4% paraformaldehyde. The brain was removed, and frozen coronal sections of 10 μm thickness were cut. The sections were blocked with 5% normal donkey serum for 1 h at room temperature. After rinsing with phosphate-buffered saline (PBS), the brain sections were incubated with the following primary antibodies: IL-1β (1:100, Abcam, Cambridge, MA, USA; cat. no. ab9787) and Iba1 (1:100, Abcam, Cambridge, MA, USA; cat. no. ab15690), caspase-3 (1:100, Cell Signaling Technology, Danvers, MA, USA; cat. no. 9664S), and NeuN (1:100, Millipore, Billerica, MA, USA; cat. no. 2766373) at 4 °C for overnight. On the following day, the sections were incubated with the secondary antibodies Alexa Fluor® 555 Donkey Anti-Rabbit IgG (H + L) (1:100, Invitrogen Life Technologies, Carlsbad, CA, USA; cat. no. A31572), Alexa Fluor® 488 donkey anti-mouse IgG (1:100, Invitrogen Life Technologies, Carlsbad, CA, USA; cat. no. A21202) for 1 h at room temperature. Finally, the sections were mounted using Fluoroshield with DAPI (Sigma, St. Louis, MO, USA; cat. no. F6057) and detected with a fluorescence microscope (Olympus DP73 Microscope, Olympus, Tokyo, Japan).

After 24 h of treatment, cover slips with adherent BV-2 cells were fixed in 4% paraformaldehyde for 20 min, blocked by 5% normal donkey serum for 30 min at room temperature. Subsequently, the cover slips were incubated at 4 °C overnight with the primary antibodies: IL-1β (1:100, Chemicon International, Temecula, CA, USA; cat. no. AB1832P), NLRP3 (1:100, Abcam, Cambridge, MA, USA; cat. no. ab4207), caspase-1 (1:100, Abcam, Cambridge, MA, USA; cat. no. ab1872). On the next day, the cover slips were incubated with the secondary antibodies Alexa Fluor® 555 Donkey Anti-Rabbit IgG (1:100, Invitrogen Life Technologie, Carlsbad, CA, USA; cat. no. A31572), Alexa Fluor® 488 Donkey Anti-Goat IgG (1:100, Abcam, Cambridge, MA, USA; cat. no. ab150129), or lectin. Finally, all cover slips were mounted using Fluoroshield with DAPI (Sigma, St. Louis, MO, USA; cat. no. F6057) and detected with a fluorescence microscope (Olympus DP73 Microscope, Olympus, Tokyo, Japan).

### Statistical analysis

The statistical analysis was performed using the SPSS 13.0. Dates are expressed as means ± standard deviation (± SD). Univariate-factor measurement data was analyzed by one-way analysis of variance (ANOVA). Repeated measurement data was analyzed by repeated-measures ANOVA. The interaction effects were analyzed by factorial ANOVA. Simple effects analyses were performed when an interaction was observed. Differences were considered statistically significant if *P* value < 0.05.

## Results

### Physiological data of rats

**Fig. 1:**
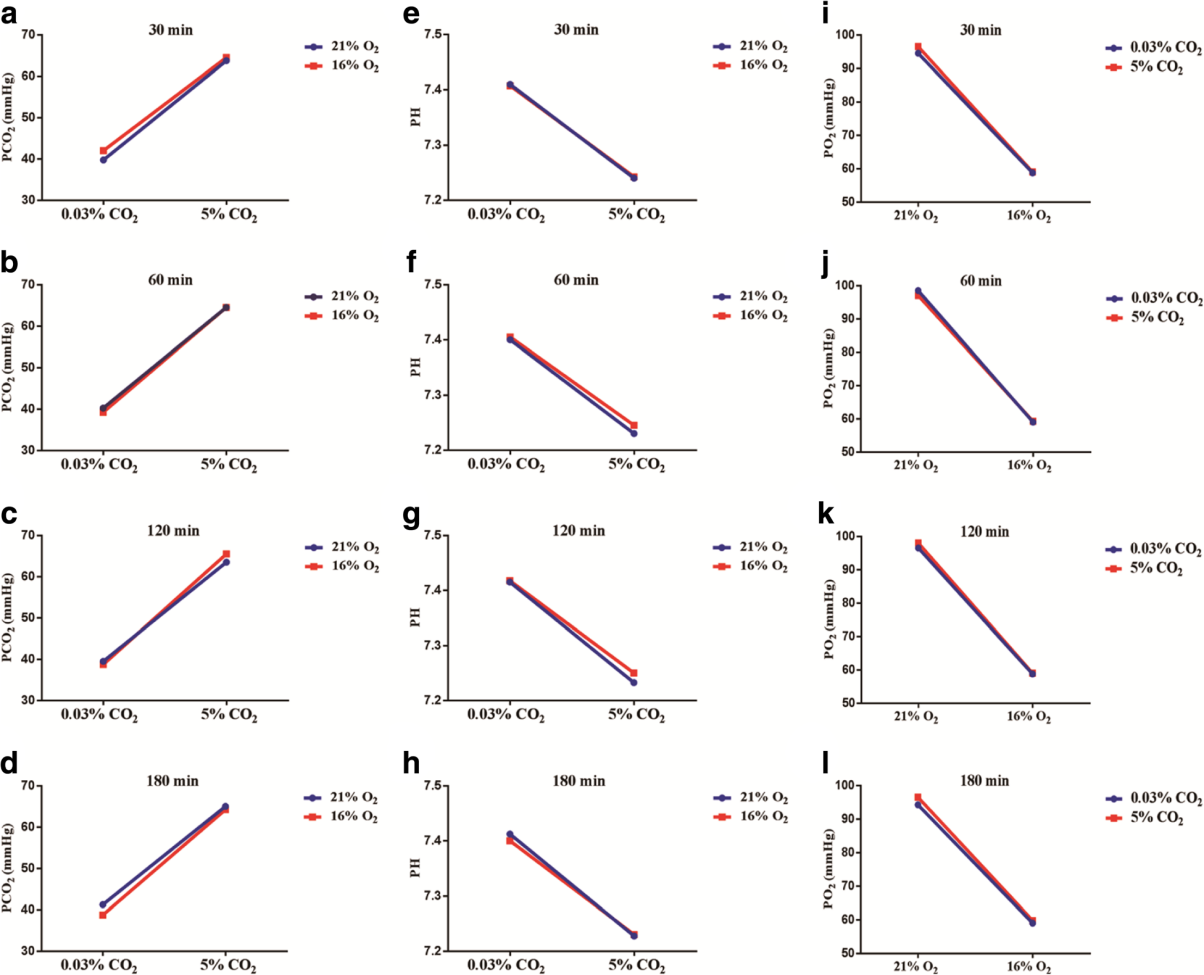
Graphs (**a**–**l**) illustrate the PaCO_2_, pH, and PO_2_ at 30, 60, 120, and 180 min after ventilation in the rats (*n* = 4). Hypercapnia (5% CO_2_) treatment significantly increases the PaCO_2_ to 60–69 mmHg, with pH at 7.20–7.25, and hypoxia treatment (16% O_2_) maintains the PO_2_ levels at around 60 mmHg, while there are no interaction effects between hypercapnia treatment and hypoxia treatment (*P* > 0.05). The concentrations of CO_2_ and O_2_ in the air are 0.03 and 21%, respectively

The PaCO_2_ levels were maintained at 35–45 mmHg in the S and hypoxemia group, and hypercapnia treatment significantly increased the PaCO_2_ to 60–69 mmHg, with pH at 7.20–7.25 in the hypercapnia and HH group. Hypercapnia treatment had main effects on elevated PaCO_2_ levels (30 min: df = 1, *F* = 280.51, *P* < 0.01; 60 min: df = 1, *F* = 389.44, *P* < 0.01; 120 min: df = 1, *F* = 483.10, *P* < 0.01; 180 min: df = 1, *F* = 500.51, *P* < 0.01) and reduced pH levels (30 min: df = 1, *F* = 260.18, *P* < 0.01; 60 min: df = 1, *F* = 272.06, *P* < 0.01; 120 min: df = 1, *F* = 364.41, *P* < 0.01; 180 min: df = 1, *F* = 544.50, *P* < 0.01) (Fig. 1a–h). The PO_2_ levels were maintained at around 60 mmHg in the hypoxemia and HH group. Hypoxia treatment had main effects on reduced PaO_2_ levels (30 min: df = 1, *F* = 157.52, *P* < 0.01; 60 min: df = 1, *F* = 261.59, *P* < 0.01; 120 min: df = 1, *F* = 275.26, *P* < 0.01; 180 min: df = 1, *F* = 347.61, *P* < 0.01) (Fig. 1i–l), while there were no interaction effects between hypercapnia treatment and hypoxia treatment (*P* > 0.05).

### Hypercapnia aggravated cognitive impairment in hypoxemic rats

**Fig. 2:**
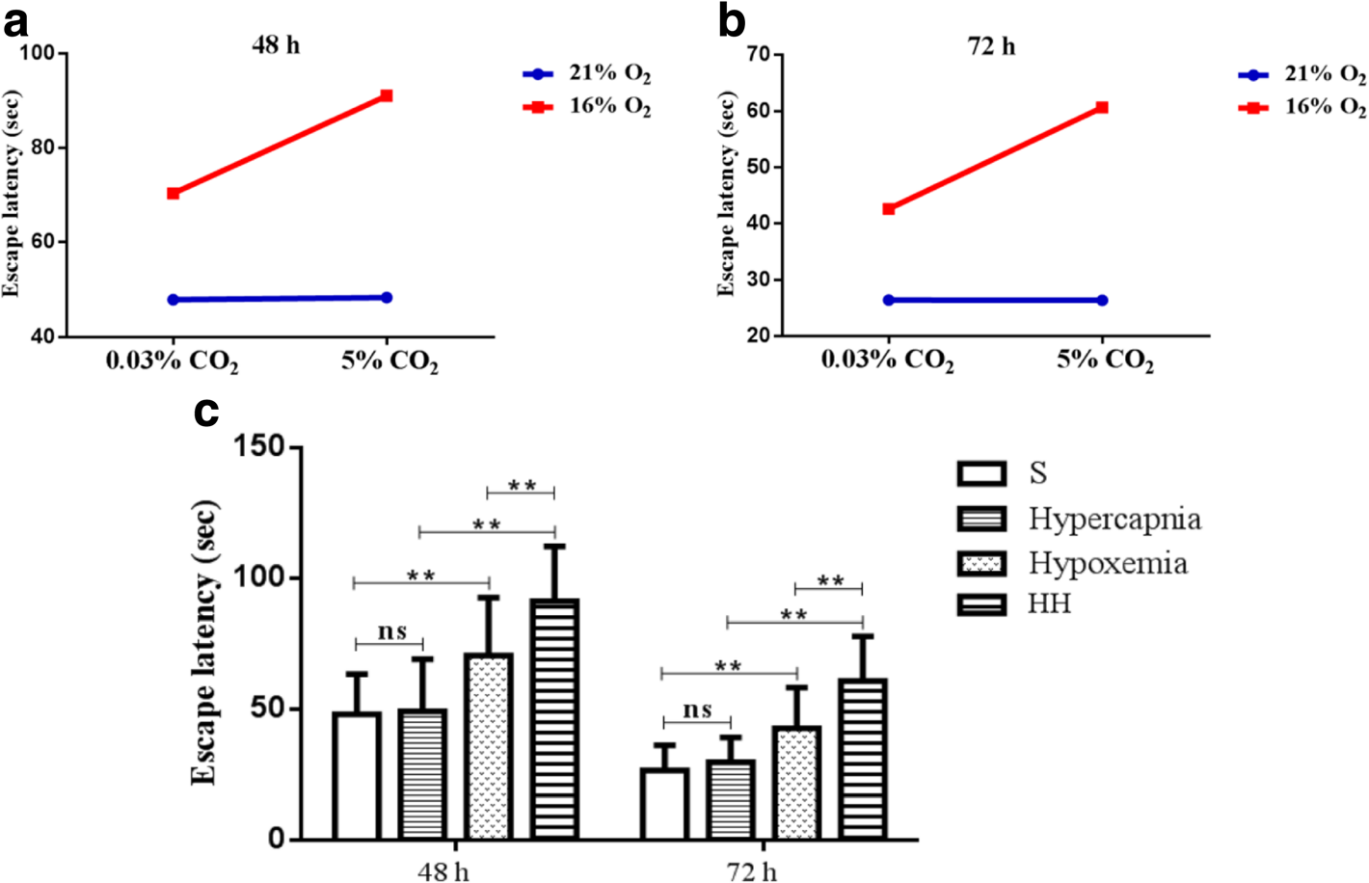
Average MWM latency time of rats in all groups (*n* = 15). **a**, **b** There are significant interaction effects between hypercapnia and hypoxia treatment at 48 and 72 h (*P* < 0.05). **c** Simple effects analyses show that there is no difference between hypercapnia group and S group at 48 and 72 h (ns *P* > 0.05). Hypoxemia group has a longer escape latency than S group at 48 and 72 h (**P < 0.01). HH group has the longest escape latency in comparison with hypercapnia group (**P < 0.01) and hypoxemia group (**P < 0.01) at 48 and 72 h. *S group* sham-operated group, *HH group* hypercapnia + hypoxemia group. The concentrations of CO_2_ and O_2_ in the air are 0.03 and 21%, respectively

To elucidate the effect of hypercapnia on cognitive function in hypoxemic rats, we conducted MWM tests. There were significant interaction effects between hypercapnia treatment and hypoxia treatment (48 h: df = 1, *F* = 4.12, *P* < 0.05; 72 h: df = 1, *F* = 6.56, *P* < 0.05) (Fig. 2a, b). In addition, simple effects analyses showed that there was no difference between hypercapnia group and S group (48 h: df = 1, *F* = 0.00, *P* > 0.05; 72 h: df = 1, *F* = 0.00, *P* > 0.05). Hypoxemia group had a longer escape latency than S group (48 h: df = 1, *F* = 10.15, *P* < 0.01; 72 h: df = 1, *F* = 10.44, *P* < 0.01). HH group had the longest escape latency in comparison with hypercapnia group (48 h: df = 1, *F* = 36.69, *P* < 0.01; 72 h: df = 1, *F* = 46.98, *P* < 0.01) and hypoxemia group (48 h: df = 1, *F* = 8.62, *P* < 0.01; 72 h: df = 1, *F* = 13.09, *P* < 0.01) (Fig. 2 c).

### Hypercapnia enhanced activation of NLRP3 inflammasome and expression of IL-1β in the hypoxic hippocampus

**Fig. 3:**
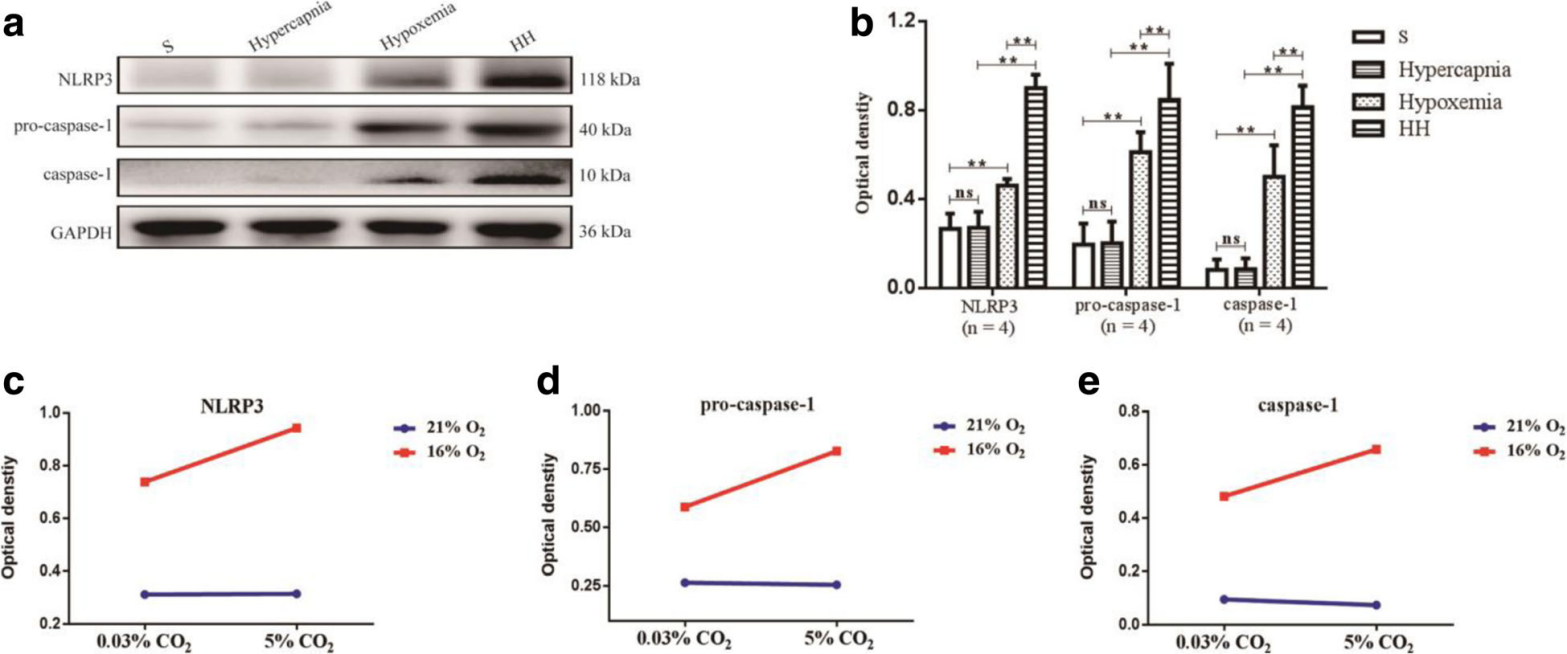
Hypercapnia enhances activation of NLRP3 inflammasome in the hypoxic hippocampus (*n* = 4). **a** The immunoreactive bands of NLRP3 (118 kDa), pro-caspase-1 (40 kDa), caspase-1 (10 kDa), and GAPDH (36 kDa). **c**–**e** There are significant interaction effects between hypercapnia treatment and hypoxia treatment (NLRP3: *P* < 0.01; pro-caspase-1: *P* < 0.01; caspase-1: *P* < 0.05). **b** Simple effects analyses show that the protein expression levels in hypercapnia group has no significant difference compared with S group (ns *P* > 0.05). The protein expression levels in hypoxemia group are significantly increased compared with S group (**P < 0.01). HH group has the highest levels of the protein in comparison with hypercapnia group (**P < 0.01) and hypoxemia group (**P < 0.01). *ns* non-significant, *S group* sham-operated group, *HH group* hypercapnia + hypoxemia group. The concentrations of CO_2_ and O_2_ in the air are 0.03 and 21%, respectively

**Fig. 4:**
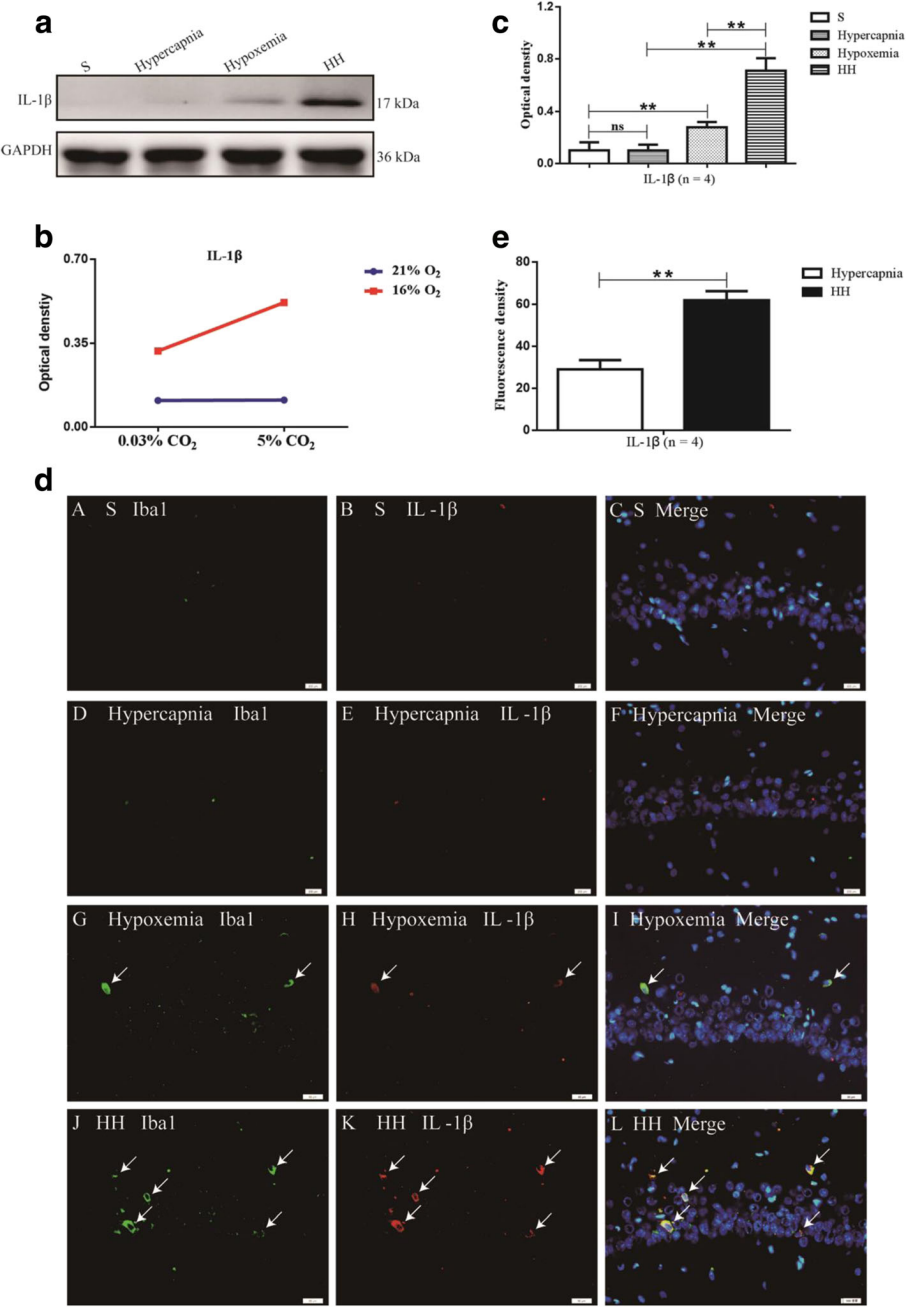
Hypercapnia enhances the release of IL-1β in the hypoxic hippocampus (*n* = 4). **a** The immunoreactive bands of IL-1β (17 kDa) and GAPDH (36 kDa). **b** There is a significant interaction effect between hypercapnia treatment and hypoxia treatment (*P* < 0.01). **c** Simple effects analyses show that the protein expression levels of IL-1β in hypercapnia group have no significant difference compared with S group (ns *P* > 0.05). The protein expression levels in hypoxemia group are significantly increased compared with S group (**P < 0.01). HH group has the highest levels of the protein in comparison with hypercapnia group (**P < 0.01) and hypoxemia group (**P < 0.01). **d** Immunofluorescence images show the expression of IL-1β (B, E, H, K, red), Iba1^+^ microglia (A, D, G, J, green), and the co-localization of IL-1β and microglia (C, F, I, L). The results also show that hypercapnia alone is not enough to increase the expression of IL-1β. The expression of IL-1β in activated microglia in the hypoxic hippocampus is markedly increased. Additionally, the expression of IL-1β is further enhanced following treatment of hypercapnia in the hypoxic hippocampus. Scale bars: (A-L), 20 μm. **e** The average fluorescence (red) density of one single microglia in hypoxemia group and HH group was analyzed by an image analysis system (Image-Pro Plus software). Statistical significance was examined by *t* test. The fluorescence density in HH group is significantly increased compared with hypoxemia group (**P < 0.01). *ns* non-significant, *IL-1β* interleukin-1 beta, *S group* sham-operated group, *HH group* hypercapnia + hypoxemia group. The concentrations of CO_2_ and O_2_ in the air are 0.03 and 21%, respectively

To explore the effects of hypercapnia on the expression of IL-1β and activation of NLRP3 inflammasome, the protein expression of IL-1β and members of NLRP3 inflammasome including NLRP3, pro-caspase-1, and caspase-1 in the hippocampus was detected by Western blot analysis. There were significant interaction effects between hypercapnia treatment and hypoxia treatment (NLRP3: df = 1, *F* = 11.54, *P* < 0.01; pro-caspase-1: df = 1, *F* = 21.55, *P* < 0.01; caspase-1: df = 1, *F* = 6.02, *P* < 0.05; IL-1β: df = 1, *F* = 12.57, *P* < 0.01) (Figs. 3c–e and 4b). In addition, simple effects analyses showed that the protein expression levels in hypercapnia group had no significant difference compared with S group (NLRP3: df = 1, *F* = 0.01, *P* > 0.05; pro-caspase-1: df = 1, *F* = 0.06, *P* > 0.05; caspase-1: df = 1, *i*= 0.16, *P* > 0.05; IL-1β: df = 1, *F* = 0.00, *P* > 0.05). The protein expression levels in hypoxemia group were significantly increased compared with S group (NLRP3: df = 1, *F* = 102.57, *P* < 0.01; pro-caspase-1: df = 1, *F* = 72.76, *P* < 0.01; caspase-1: df = 1, *F* = 51.48, *P* < 0.01; IL-1β: df = 1, *F* = 26.50, *P* < 0.01). HH group had the highest levels of the protein in comparison with hypercapnia group (NLRP3: df = 1, *F* = 222.94, *P* < 0.01; pro-caspase-1: df = 1, F = 227.86, *P* < 0.01; caspase-1: df = 1, *F* = 117.93, *P* < 0.01; IL-1β: df = 1, *F* = 103.25, *P* < 0.01) and Hypoxemia group (NLRP3: df = 1, *F* = 23.81, *P* < 0.01; pro-caspase-1: df = 1, *F* = 40.04, *P* < 0.01; caspase-1: df = 1, *F* = 10.77, *P* < 0.01; IL-1β: df = 1, F = 25.62, *P* < 0.01) (Figs. 3a, b and 4a, c).

### Hypercapnia increased the expression of IL-1β in activated microglia in the hypoxic hippocampus

To investigate whether hypercapnia would increase the expression of IL-1β in microglia, IL-1β in the microglia in hippocampus CA1 area was examined by double immunofluorescence. Hypercapnia alone is not enough to cause immunofluorescence enhancement of IL-1β. The immunofluorescence of IL-1β in hypoxia-activated microglia in the hippocampus was noticeably enhanced. Additionally, the immunofluorescence was further enhanced following treatment of hypercapnia in the hypoxic hippocampus (Fig. 4d). The average fluorescence density of one single microglia in hypoxemia group and HH group was analyzed by an image analysis system (Image-Pro Plus software). The fluorescence density in HH group was significantly increased compared with hypoxemia group (*P* < 0.01) (Fig. 4e).

### Hypercapnia aggravated apoptosis of hippocampal neurons of hypoxemic rats

**Fig. 5:**
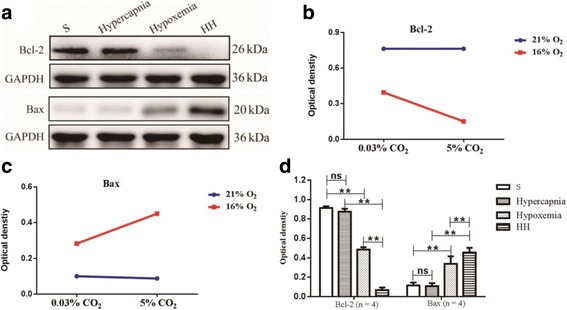
Hypercapnia aggravates apoptosis of hippocampal neurons of hypoxemic rats (*n* = 4). **a** The immunoreactive bands of Bcl-2 (26 kDa), Bax (20 kDa), and GAPDH (36 kDa). **b**, **c** There are significant interaction effects between hypercapnia treatment and hypoxia treatment (Bcl-2: *P* < 0.01; Bax: *P* < 0.05). **d** Simple effects analyses show that the protein expression levels of Bcl-2 and Bax in hypercapnia group have no significant difference compared with S group (ns *P* > 0.05). The protein expression levels of Bcl-2 in hypoxemia group are significantly decreased compared with S group (**P < 0.01). HH group has the lowest levels of the protein in comparison with hypercapnia group (**P < 0.01) and hypoxemia group (**P < 0.01). In contrast, a significant increase in Bax expression is observed in hypoxemia group compared with S group (**P < 0.01). The levels of Bax expression in HH group is the highest as compared to hypercapnia group (**P < 0.01) and hypoxemia group (**P < 0.01). *S group* sham-operated group, *HH group* hypercapnia + hypoxemia group. The concentrations of CO_2_ and O_2_ in the air are 0.03 and 21%, respectively

**Fig. 6:**
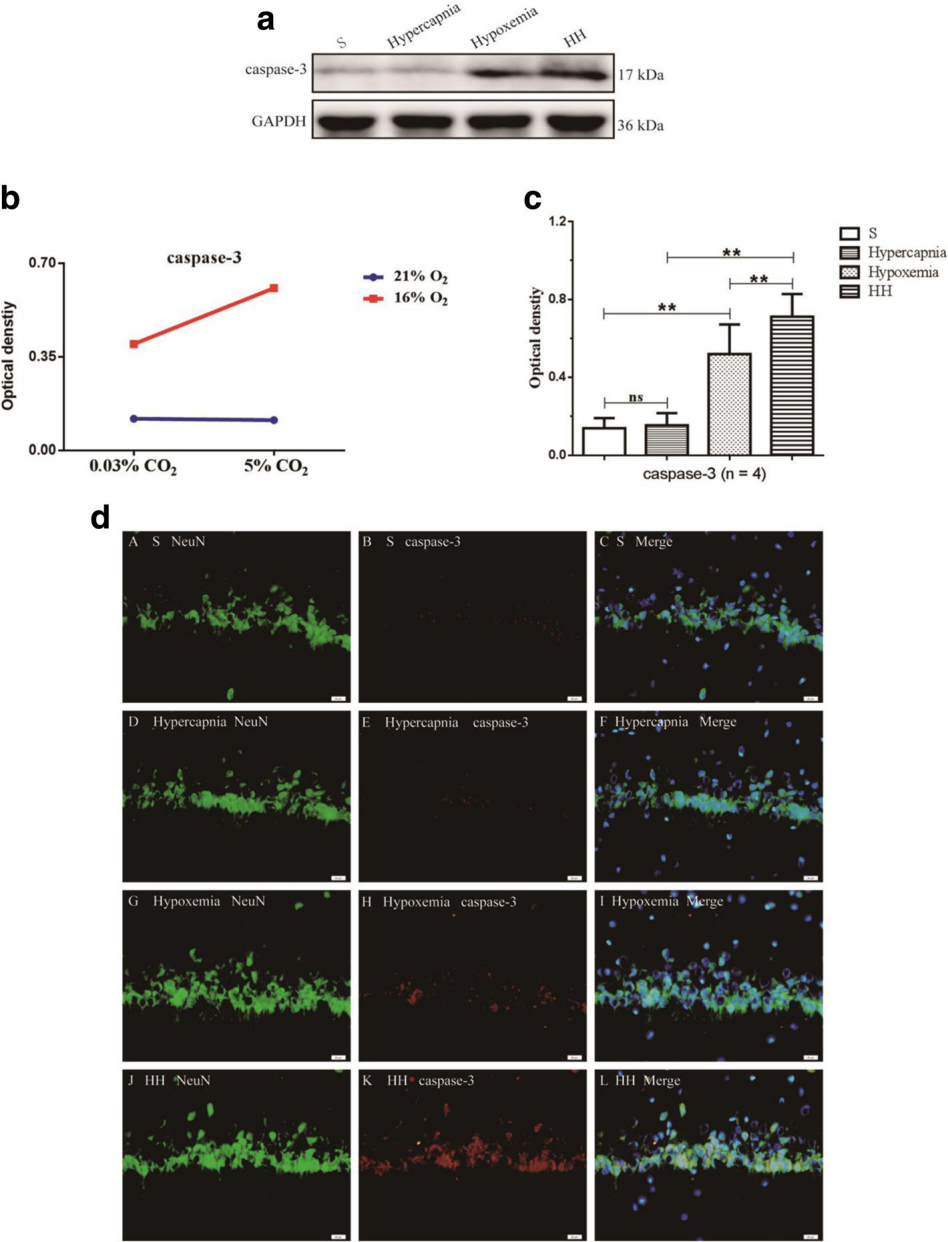
Hypercapnia aggravates apoptosis of hippocampal neurons of hypoxemic rats (*n* = 4). **a** The immunoreactive bands of caspase-3 (17 kDa) and GAPDH (36 kDa). **b** There is a significant interaction effect between hypercapnia treatment and hypoxia treatment (*P* < 0.05). **c** Simple effects analyses show that the protein expression levels of caspase-3 in hypercapnia group have no significant difference compared with S group (ns *P* > 0.05). A significant increase in caspase-3 expression is observed in hypoxemia group compared with S group (**P < 0.01). The levels of caspase-3 expression in HH group is the highest as compared to hypercapnia group (**P < 0.01) and hypoxemia group (**P < 0.01). **d** Immunofluorescence images show the expression of caspase-3 (B, E, H, K, red), NeuN^+^ neurons (A, D, G, J, green), and the co-localization of caspase-3 and neurons (C, F, I, L). The results also show that hypercapnia alone is not enough to increase the expression of caspase-3. The expression of caspase-3 in neurons in the hypoxic hippocampus is markedly increased. Additionally, the expression of caspase-3 is further enhanced following treatment of hypercapnia in the hypoxic hippocampus. Scale bars: (A-L), 20 μm. *S group* sham-operated group, *HH group* hypercapnia + hypoxemia group. The concentrations of CO_2_ and O_2_ in the air are 0.03 and 21%, respectively

To determine if hypercapnia played any role in apoptosis of hippocampal neurons in hypoxemic rats, the expression levels of Bcl-2, Bax, and caspase-3 were examined by Western blot analysis. There were significant interaction effects between hypercapnia treatment and hypoxia treatment (Bcl-2: df = 1, *F* = 9.73, *P* < 0.01; Bax: df = 1, *F* = 6.26, *P* < 0.05; caspase-3: df = 1, *F* = 7.74, *P* < 0.05) (Figs. 5b, c and 6b). In addition, simple effects analyses showed that the protein expression of Bcl-2, Bax, and caspase-3 in hypercapnia group had no significant difference compared with S group (Bcl-2: df = 1, *F* = 0.00, *P* > 0.05; Bax: df = 1, *F* = 0.05, *P* > 0.05; caspase-3: df = 1, *F* = 0.01, *P* > 0.05). The protein expression levels of Bcl-2 in hypoxemia group were significantly decreased compared with S group (df = 1, *F* = 43.77, *P* < 0.01). HH group had the lowest levels of the protein in comparison with hypercapnia group (df = 1, *F* = 121.59, *P* < 0.01) and hypoxemia group (df = 1, *F* = 19.45, *P* < 0.01). In contrast, a significant increase in Bax and caspase-3 expression was observed in hypoxemia group compared with S group (Bax: df = 1, *F* = 39.67, *P* < 0.01; caspase-3: df = 1, *F* = 26.24, *P* < 0.01). The levels of Bax and caspase-3 expression in HH group were the highest as compared to hypercapnia group (Bax: df = 1, *F* = 96.77, *P* < 0.01; caspase-3: df = 1, *F* = 82.02, *P* < 0.01) and hypoxemia group (Bax: df = 1, *F* = 11.06, *P* < 0.01; caspase-3: df = 1, *F* = 14.76, *P* < 0.01) (Figs. 5a, d and 6a, c).

To investigate whether hypercapnia would aggravate apoptosis of hippocampal neurons, expression of caspase-3 in the neurons in hippocampus CA1 area was examined by double immunofluorescence. Hypercapnia alone was not enough to cause immunofluorescence enhancement of caspase-3. The immunofluorescence in neurons in the hypoxic hippocampus was noticeably enhanced. Additionally, the immunofluorescence was further augmented following hypercapnia in the hypoxic hippocampus (Fig. 6d).

### PO_2_, PCO_2_, and pH of supernatants

**Fig. 7:**
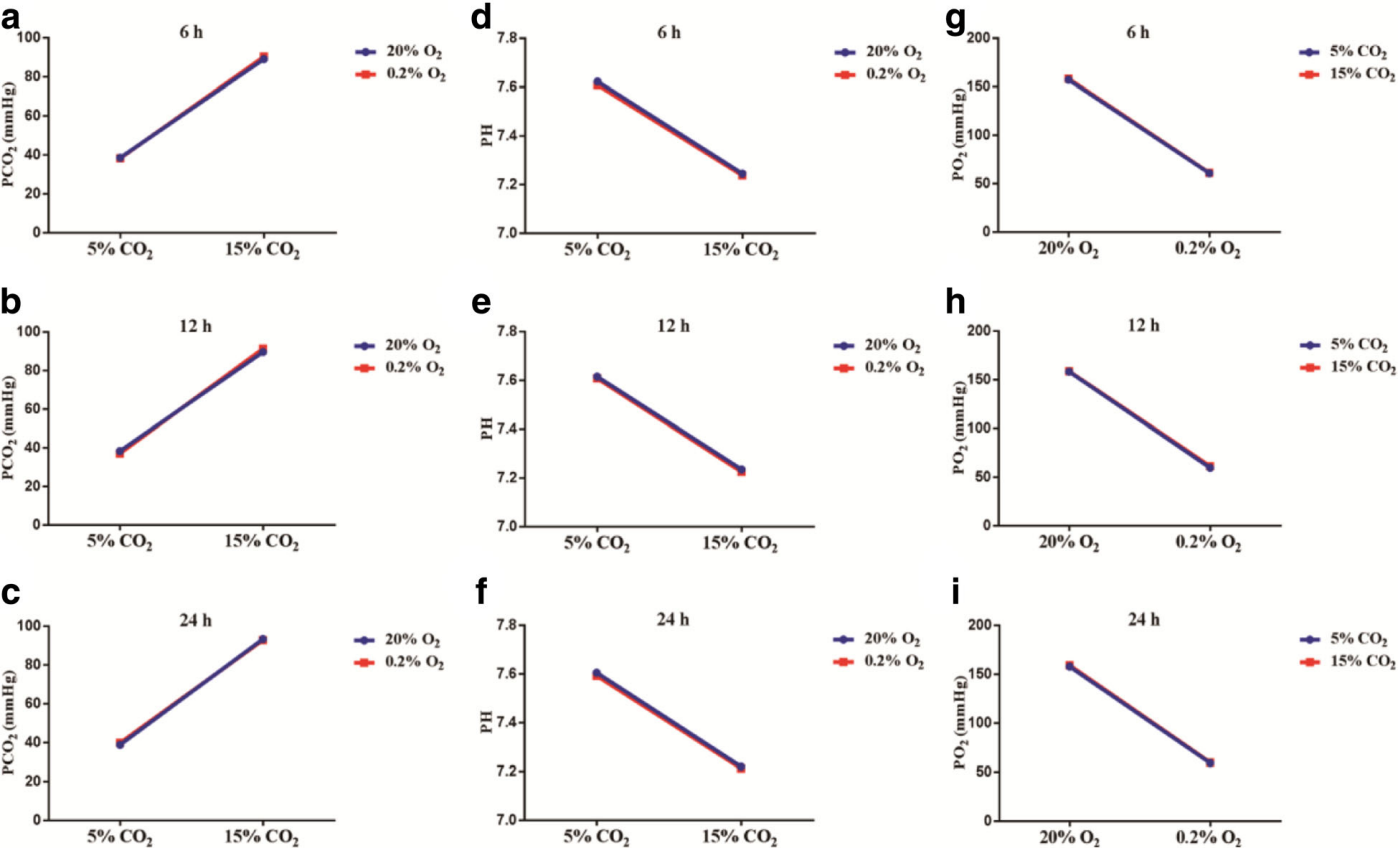
Graphs **a** to **i** illustrate the PaCO_2_, pH, and PO_2_ of supernatants (*n* = 4). 15% CO_2_ treatment significantly increases the PaCO_2_, with pH at 7.20–7.25, and 0.2% O_2_ treatment maintains the PO_2_ levels at around 60 mmHg, while there are no interaction effects between 15% CO_2_ treatment and 0.2% O_2_ treatment (*P* > 0.05)

The PaCO_2_ levels were maintained at 35–45 mmHg in the control and hypoxia group, and 15% CO_2_ treatment significantly increased the PaCO_2_, with pH at 7.20–7.25 in the HC and hypoxia + HC group. 15% CO_2_ treatment had main effects on elevated PaCO_2_ levels (6 h: df = 1, *F* = 2966.47, *P* < 0.01; 12 h: df = 1, *F* = 3469.17, *P* < 0.01; 24 h: df = 1, *F* = 3594.06, *P* < 0.01) and reduced pH levels (6 h: df = 1, *F* = 2035.57, *P* < 0.01; 12 h: df = 1, *F* = 3207.76, *P* < 0.01; 24 h: df = 1, *F* = 3483.16, *P* < 0.01) (Fig. 7a–f). The PaO_2_ values were maintained at around 60 mmHg in the hypoxia and hypoxia + HC group. 0.2% O_2_ treatment had main effects on reduced PaO_2_ levels (6 h: df = 1, *F* = 8342.41, *P* < 0.01; 12 h: df = 1, *F* = 10,597.52, *P* < 0.01; 24 h: df = 1, *F* = 14,040.21, *P* < 0.01) (Fig. 7g–i), while there were no interaction effects between 15% CO_2_ treatment and 0.2% O_2_ treatment (*P* > 0.05).

### Higher levels of CO_2_ treatment time-dependently increased protein expression of IL-1β in hypoxic BV-2 cells

**Fig. 8:**
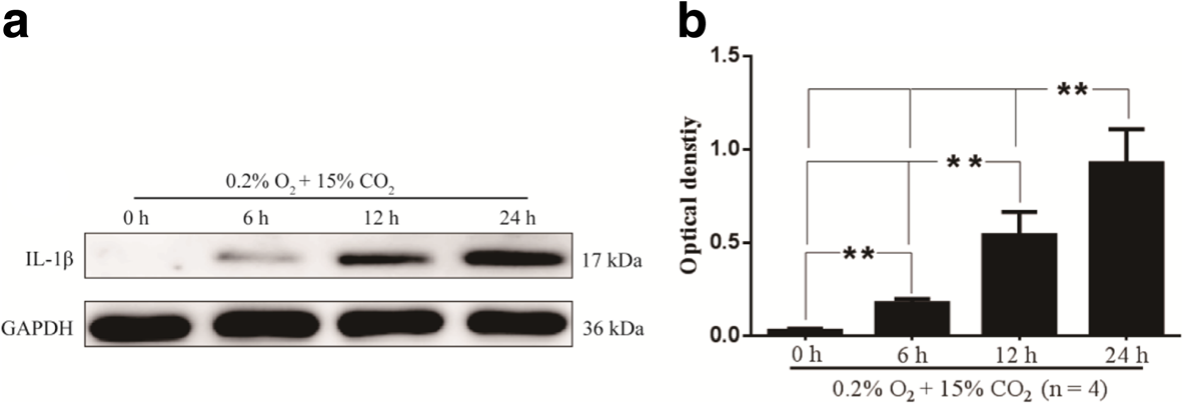
Higher levels of CO_2_ treatment time-dependently increase protein expression of IL-1β in hypoxic BV-2 cells (*n* = 4). **a** The immunoreactive bands of IL-1β (17 kDa) and GAPDH (36 kDa). Bar graph **b** shows the protein expression of IL-1β in hypoxic BV-2 cells is increased with the prolonging of time, and there is significant difference among each group (*P < 0.01). *IL-1β* interleukin-1 beta

To determine the optimal time-point of intervention for the cells, the protein expression of IL-1β in the BV-2 cells was examined by Western blot at 6, 12, and 24 h. 15% CO_2_ treatment increased the protein expression of IL-1β in hypoxic BV-2 cells with the prolonging of time; there was significant difference among each group (df = 3, *F* = 42.22, *P* < 0.01). In view of this, the time-point 24 h was chosen as the intervention time in the follow-up in vitro experiment (Fig. 8a, b).

### High concentration of CO_2_ enhanced activation of NLRP3 inflammasome coupled with increased IL-1β expression in hypoxic BV-2 microglia

**Fig. 9:**
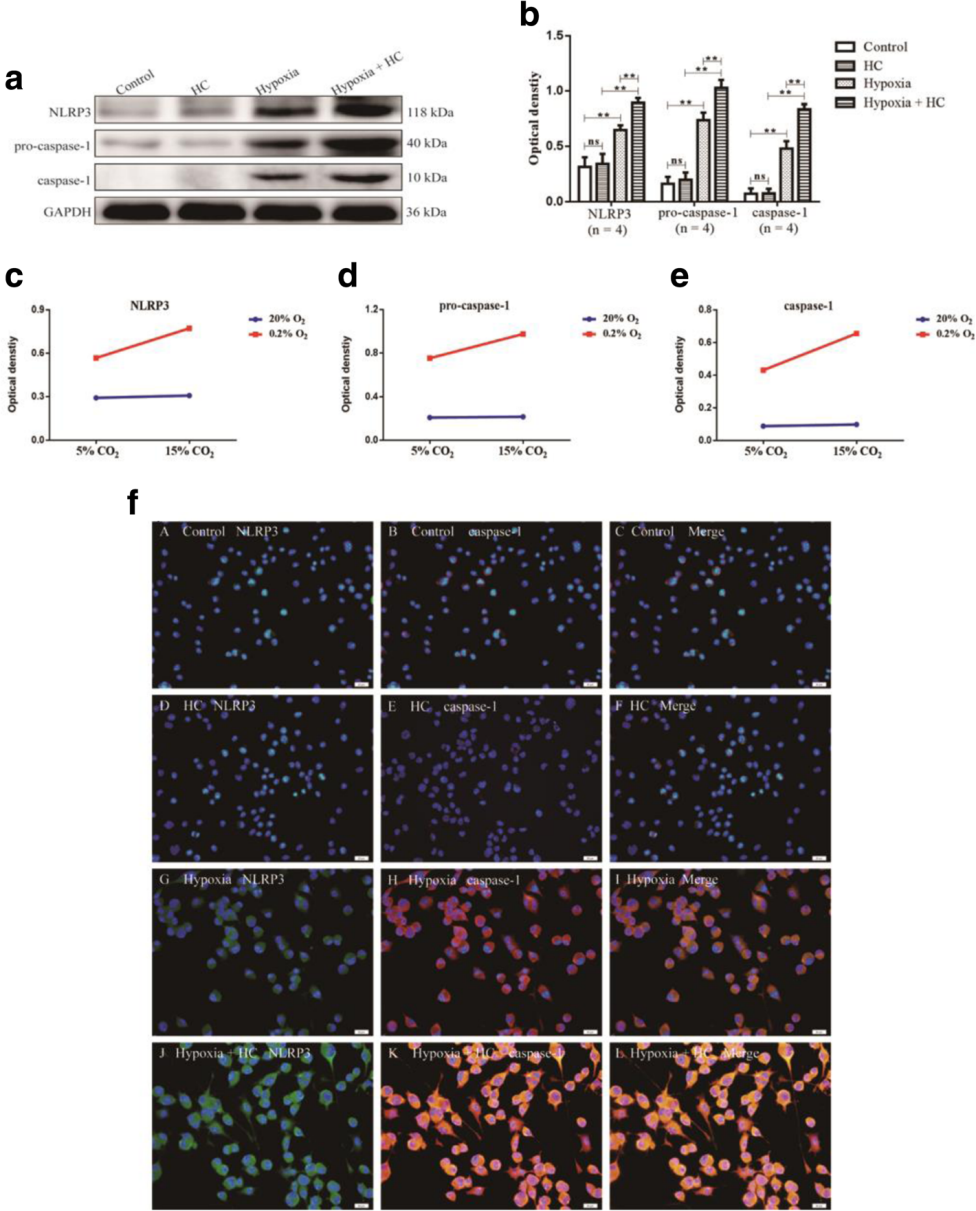
High concentration of CO_2_ enhances activation of NLRP3 inflammasome in hypoxic BV-2 microglia (*n* = 4). **a** The immunoreactive bands of NLRP3 (118 kDa), pro-caspase-1 (40 kDa), caspase-1 (10 kDa), and GAPDH (36 kDa). **c**–**e** There are significant interaction effects between 15% CO_2_ treatment and 0.2% O_2_ treatment (NLRP3: *P* < 0.01; pro-caspase-1: *P* < 0.01; caspase-1: *P* < 0.05). **b** Simple effects analyses show that the protein expression in HC group has no significant difference compared with control group (ns *P* > 0.05). The protein expression levels in hypoxia group are significantly increased compared with control group (**P < 0.01). Hypoxia + HC group has the highest levels of the protein in comparison with HC group (**P < 0.01) and hypoxia group (**P < 0.01). **f** Immunofluorescence images show the expression of caspase-1 (B, E, H, K, red), NLRP3 (A, D, G, J, green), and the co-localization of caspase-1 and NLRP3 (C, F, I, L). The results also show that high concentration of CO_2_ (15% CO_2_) alone is not enough to increase the expression of caspase-1 and NLRP3. The expression of caspase-1 and NLRP3 in hypoxia-activated BV-2 microglia is markedly increased. Additionally, the expression of caspase-1 and NLRP3 is further enhanced following treatment of 15% CO_2_ in the hypoxic BV-2 microglia. Scale bars: (A-L), 20 μm. *HC group* high concentration of carbon dioxide group

**Fig. 10:**
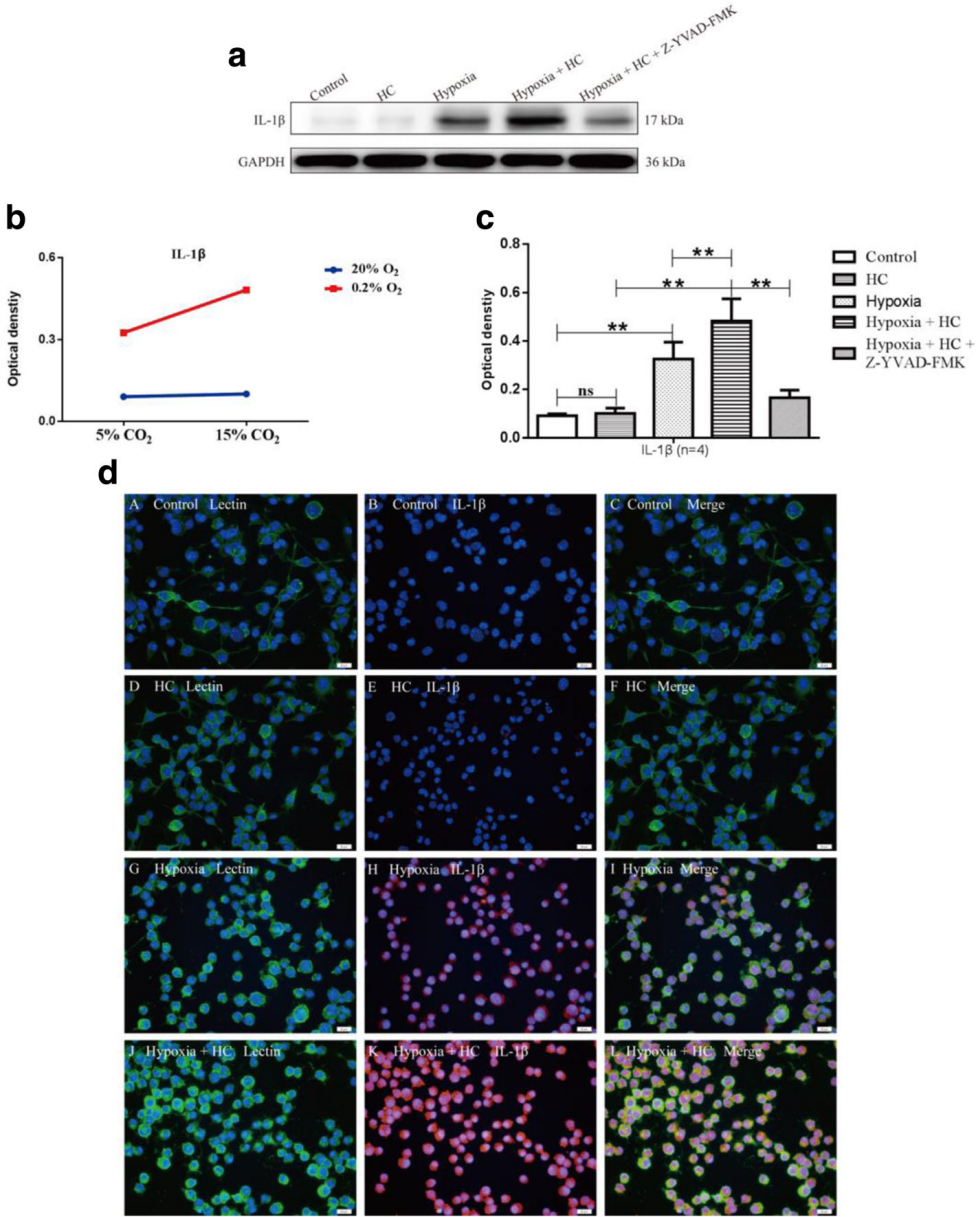
High concentration of CO_2_ enhances the expression of IL-1β in hypoxic BV-2 microglia (*n* = 4). **a** The immunoreactive bands of IL-1β (17 kDa) and GAPDH (36 kDa). **b** There is a significant interaction effect between 15% CO_2_ treatment and 0.2% O_2_ treatment (*P* < 0.05). **c** Simple effects analyses show that the protein expression of IL-1β in HC group has no significant difference compared with Control group (ns *P* > 0.05). The protein expression levels in hypoxia group are significantly increased compared with control group (**P < 0.01). Hypoxia + HC group had the highest levels of the protein in comparison with HC group (**P < 0.01) and hypoxia group (** *P* < 0.01). The protein expression of IL-1β is significantly suppressed with the treatment of 10 μM Z-YVAD-FMK (**P < 0.01). **d** Immunofluorescence images show the expression of IL-1β (B, E, H, K, red), lectin^+^ microglia (A, D, G, J, green), and the co-localization of IL-1β and microglia (C, F, I, L). The results also show that high concentration of CO_2_ (15% CO_2_) alone is not enough to increase the expression of IL-1β. The expression of IL-1β in hypoxia-activated BV-2 microglia is markedly increased. Additionally, the expression of IL-1β is further enhanced following treatment of 15% CO_2_ in the hypoxic BV-2 microglia. Scale bars: (A-L), 20 μm. *ns* non-significant, *IL-1β* interleukin-1 beta, *HC group* high concentration of carbon dioxide group

To explore the effects of high concentration of CO_2_ on activation of NLRP3 inflammasome and the expression of IL-1β, the protein expression of IL-1β, NLRP3, pro-caspase-1, and caspase-1 in BV-2 microglia was detected by Western blot. There were significant interaction effects between 15% CO_2_ treatment and 0.2% O_2_ treatment (NLRP3: df = 1, *F* = 11.25, *P* < 0.01; pro-caspase-1: df = 1, *F* = 10.60, *P* < 0.01; caspase-1: df = 1, *F* = 7.47, *P* < 0.05; IL-1β: df = 1, *F* = 6.24, *P* < 0.05) (Figs. 9c–e and 10b). In addition, simple effects analyses showed that the protein expression in HC group had no significant difference compared with control group (NLRP3: df = 1, *F* = 0.14, *P* > 0.05; pro-caspase-1: df = 1, *F* = 0.03, *P* > 0.05; caspase-1: df = 1, *F* = 0.03, *P* > 0.05; IL-1β: df = 1, *F* = 0.06, *P* > 0.05). The protein expression levels in hypoxia group were significantly increased compared with control group (NLRP3: df = 1, *F* = 47.14, *P* < 0.01; pro-caspase-1: df = 1, *F* = 56.88, *P* < 0.01; caspase-1: df = 1, *F* = 37.92, *P* < 0.01; IL-1β: df = 1, *F* = 31.91, *P* < 0.01). Hypoxia + HC group had the highest levels of the protein in comparison with HC group (NLRP3: df = 1, *F* = 134.79, *P* < 0.01; pro-caspase-1: df = 1, *F* = 212.38, *P* < 0.01; caspase-1: df = 1, *F* = 100.46, *P* < 0.01; IL-1β: df = 1, *F* = 84.31, *P* < 0.01) and hypoxia group (NLRP3: df = 1, *F* = 26.20, *P* < 0.01; pro-caspase-1: df = 1, *F* = 51.86, *P* < 0.01; caspase-1: df = 1, *F* = 16.36, *P* < 0.01; IL-1β: df = 1, *F* = 14.33, *P* < 0.01). The protein expression of IL-1β was markedly enhanced by high concentration of CO_2_ in hypoxic BV-2 microglia; it was significantly suppressed with the treatment of 10 μM Z-YVAD-FMK (*P* < 0.01) (Figs. 9a, b and 10a, c).

High concentration of CO_2_ is not enough to cause immunofluorescence enhancement of NLRP3, caspase-1, and IL-1β in BV-2 microglia without hypoxia. However, the immunofluorescence in hypoxia-activated BV-2 microglia was noticeably enhanced. Additionally, the immunofluorescence was further enhanced following treatment of high concentration of CO_2_ in hypoxia-activated BV-2 microglia (Figs. 9f and 10d).

### High concentration of CO_2_ aggravated apoptosis of cultured neurons via hypoxia-activated microglia

**Fig. 11:**
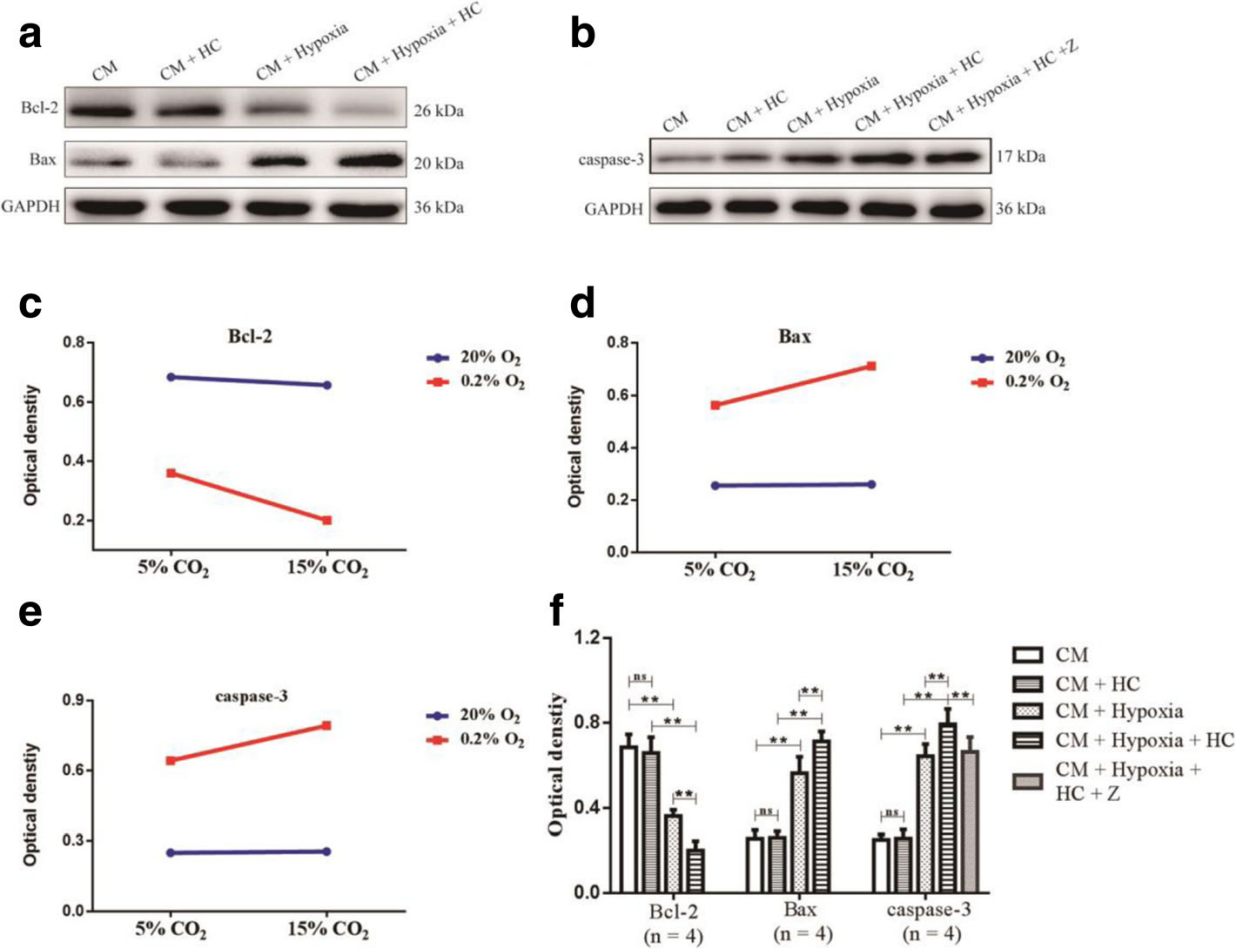
High concentration of CO_2_ aggravates apoptosis of cultured neurons via hypoxia-activated microglia (*n* = 4). **a**, **b** The immunoreactive bands of Bcl-2 (26 kDa), Bax (20 kDa), caspase-3 (17 kDa), and GAPDH (36 kDa). **c**–**e** There are significant interaction effects between CM + HC treatment and CM + hypoxia treatment (Bcl-2: *P* < 0.05; Bax: *P* < 0.05; caspase-3: *P* < 0.05). **f** Simple effects analyses show that the protein expression of Bcl-2, Bax, and caspase-3 in CM + HC group has no significant difference compared with CM group (ns *P* > 0.05). The protein expression levels of Bcl-2 in CM + hypoxia group are significantly decreased compared with CM group (**P < 0.01). CM + hypoxia + HC group has the lowest levels of the protein in comparison with CM + HC group (**P < 0.01) and CM + hypoxia group (**P < 0.01). In contrast, a significant increase in Bax and caspase-3 expression is observed in CM + hypoxia group compared with CM group (**P < 0.01). The levels of Bax and caspase-3 expression in CM + hypoxia + HC group is the highest as compared to CM + HC group (**P < 0.01) and CM + hypoxia group (**P < 0.01). When BV-2 conditioned medium is pretreated with Z-YVAD-FMK, the protein expression of caspase-3 of neurons is significantly suppressed (**P < 0.01). *CM* conditioned medium, *HC* high concentration of carbon dioxide, *Z* Z-YVAD-FMK

To determine if high concentration of CO_2_ played any role in apoptosis of cultured neurons via hypoxia-activated microglia, cultured microglia cells were stimulated with 0.2% O_2_ or (and) 15% CO_2_, and conditioned medium derived from those cultures was used to maintain neuronal cultures. The expression levels of Bcl-2, Bax, and caspase-3 of neurons were examined by Western blot analysis. There were significant interaction effects between CM + HC treatment and CM + hypoxia treatment (Bcl-2: df = 1, *F* = 5.89, *P* < 0.05; Bax: df = 1, *F* = 8.02, *P* < 0.05; caspase-3: df = 1, *F* = 7.68, *P* < 0.05) (Figs. 11c–e). In addition, simple effects analyses showed that the protein expression of Bcl-2, Bax, and caspase-3 in CM + HC group had no significant difference compared with CM group (Bcl-2: df = 1, *F* = 0.51, *P* > 0.05; Bax: df = 1, *F* = 0.02, *P* > 0.05; caspase-3: df = 1, *F* = 0.02, *P* > 0.05). The protein expression levels of Bcl-2 in CM + hypoxia group were significantly decreased compared with CM group (df = 1, *F* = 70.86, *P* < 0.01). CM + hypoxia + HC group had the lowest levels of the protein in comparison with CM + HC group (df = 1, *F* = 140.42, *P* < 0.01) and CM + hypoxia group (df = 1, *F* = 17.17, *P* < 0.01). In contrast, a significant increase in Bax and caspase-3 expression was observed in CM + hypoxia group compared with CM group (Bax: df = 1, *F* = 72.16, *P* < 0.01; caspase-3: df = 1, *F* = 112.55, *P* < 0.01). The levels of Bax and caspase-3 expression in CM + hypoxia + HC group were the highest as compared to CM + HC group (Bax: df = 1, *F* = 156.25, *P* < 0.01; caspase-3: df = 1, *F* = 211.07, *P* < 0.01) and CM + hypoxia group (Bax: df = 1, *F* = 17.17, *P* < 0.01; caspase-3: df = 1, *F* = 16.44, *P* < 0.01). When BV-2 conditioned medium was pretreated with Z-YVAD-FMK, the protein expression of caspase-3 of neurons was significantly suppressed (*P* < 0.01) (Figs. 11a–f).

## Discussion

In the present study, we have found that hypercapnia alone is not sufficient to induce cognitive impairment, but hypercapnia can significantly aggravate the cognitive function of hypoxic rats. In the MWM test, rats treated with hypercapnia + hypoxia had significantly longer escape latency than those treated with hypoxia. Additionally, hypercapnia can enhance activation of NLRP3 inflammasome and production of IL-1β as manifested by the increased protein expression of NLRP3, caspase-1, and IL-1β in hypoxic hippocampus and hypoxia-activated BV-2 cells. There were also interaction effects on cognitive impairment, activation of NLRP3 inflammasome, and the upregulation of IL-1β between hypercapnia treatment and hypoxia treatment.

With relatively fixed ventilator settings, when rats receive mechanical ventilation with O_2_ concentration of 16%, PaO_2_ hovered around 60 mmHg. This is consistent with the change of hypoxemia in ARDS. When rats receiving mechanical ventilation with CO_2_ concentrations of 5%, PaCO_2_ was maintained at 60–69 mmHg, with pH at 7.20–7.25 which is consistent with the change of permissive hypercapnia in ARDS. Although the optimal PaCO_2_ level is still controversial, in the absence of raised intracranial pressure and/or right cardiac failure, it has been found that PaCO_2_ up to 70 mmHg with a pH of 7.20 is safe [27, 28]. The survey showed that most of the physicians prefer to maintain an arterial pH between 7.21 and 7.25 [29].

Hippocampus is known to be critical for spatial and contextual memory [30]. Thus, Western blot or immunofluorescence staining were used to determine the expression levels of IL-1β, NLRP3, and caspase-1 in the hippocampus in each group. We show here that hypercapnia alone is not sufficient to induce IL-1β production, but NLRP3 inflammasome in hypoxic rat hippocampus microglia can be activated by hypercapnia, as evidenced by the upregulation of caspase-1 and IL-1β. Double immunofluorescence staining has further demonstrated that IL-1β expression was localized in microglia in the hippocampal CA1 region as verified by its co-localization with Iba1, a cellular marker for microglia. In light of the above, it is suggested that hypercapnia can increase the secretion of IL-1β through activating the NLRP3 inflammasome in the hypoxic hippocampal microglia.

To determine the level of apoptosis in hippocampal neurons, Bcl-2, Bax, and caspase-3 expression was examined by Western blot or double immunofluorescence staining. Along with the overexpression of IL-1β, apoptosis of hippocampal neurons increased. There was also an interaction effect on apoptosis of hippocampal neurons between hypercapnia treatment and hypoxia treatment. There was an apparent upregulation of Bax and caspase-3, but a downregulation of Bcl-2 in the hippocampus in rats treated with hypercapnia + hypoxia compared with those treated with hypoxia alone. Double immunofluorescence staining has demonstrated that caspase-3 expression was localized in neurons in the hippocampal CA1 region as verified by its co-localization with NeuN, a cellular marker for neurons. These results indicated that hypercapnia can aggravate the apoptosis of hypoxic hippocampus neurons.

In vitro results were consistent with in vivo experiments. When microglial cells were exposed to O_2_ concentrations of 0.2%, PaO_2_ in supernatants hovered around 60 mmHg. When exposed to CO_2_ concentrations of 15%, pH was maintained at 7.20–7.25. There were interaction effects on IL-1β production and activation of NLRP3 inflammasome between 15% CO_2_ treatment and 0.2% O_2_ treatment. The protein levels of NLRP3, caspase-1, and IL-1β were significantly increased by 0.2% O_2_ + 15% CO_2_ treatment compared with 0.2% O_2_ treatment by Western blot and double immunofluorescence study. However, the production of IL-1β was markedly reduced after treatment with 10 μM Z-YVAD-FMK, a caspase-1 inhibitor. These results suggest that high concentrations of CO_2_ can exert an effect in increasing IL-1β production via activating the NLRP3 inflammasome in hypoxia-activated microglia.

To determine if high concentration of CO_2_ played any role in apoptosis of neurons via hypoxia-activated microglia. Microglia-conditioned medium was used to treat cultured neurons. The expression levels of Bcl-2, Bax, and caspase-3 of neurons were examined by Western blot analysis. There was an interaction effect on apoptosis of primary neurons between high CM + HC treatment and CM + hypoxia treatment. There was an apparent upregulation of Bax and caspase-3, but a downregulation of Bcl-2 in the neurons treated with CM + hypoxia + HC compared with those treated with CM + hypoxia. However, when BV-2 conditioned medium was pretreated with Z-YVAD-FMK (a caspase-1 inhibitor), the production of caspase-3 was markedly reduced. This indicated that inhibition or suppression of NLRP3 inflammasome activation and release of IL-1β might ameliorate apoptosis of neurons. It has been reported that IL-1β can promote apoptosis and contribute to caspase-3 activation [31–34]. In light of present finding, it is suggested that the cascade of IL-1β secretion induced by high concentration of CO_2_ in microglia may be a risk factor for apoptosis of neurons.

In conclusion, this study has demonstrated for the first time that hypercapnia, besides hypoxia, functions as a modulator of inflammation of the CNS. The present results indicate that hypercapnia-induced IL-1β overproduction by hypoxia-activated microglia can exacerbate neuroinflammation as evident by the increase in neuronal death in the hippocampus. It is conceivable that this would ultimately lead or contribute to the pathogenesis of cognitive impairments. In this regard, hypercapnia induces the secretion of IL-1β via increased activation of caspase-1 specifically in hypoxia-activated microglia. Supporting this argument is the fact that production of IL-1β is attenuated by inhibiting caspase-1 activation. Thus, the cascade of hypercapnia-induced IL-1β secretion in microglia may be a potential target for treating cognitive dysfunction.

## Conclusions

We show here that hypercapnia can significantly aggravate the cognitive function of hypoxic adult rats. Remarkably, hypercapnia can enhance the activation of NLRP3 inflammasome and release of IL-1β in the hypoxia-activated microglia. Furthermore, the levels of Bcl-2 were reduced, while that of Bax and caspase-3 were increased in the hippocampal neurons by hypercapnia. Additionally, we have shown that pharmacological inhibition of NLRP3 inflammasome activation and release of IL-1β might ameliorate apoptosis of neurons. In consideration of the present results along with others, it is suggested that hypercapnia-induced IL-1β overproduction via activating the NLRP3 inflammasome by hypoxia-activated microglia may exacerbate neuroinflammation, increase neuronal death, and contribute to the pathogenesis of cognitive impairments.

## Abbreviations

ARDS: Acute respiratory distress syndrome
ASC: Apoptosis-associated speck-like protein containing a CARD
CNS: Central nervous system
CO_2_: Carbon dioxide
IL-1β: Interleukin-1 beta
MWM: Morris water maze
N_2_: Nitrogen
NLRP3: NLR family, pyrin domain-containing 3
O_2_: Oxygen
PaCO_2_: Partial pressure of carbon dioxide
PaO_2_: Artial pressure of oxygen
ROS: Reactive oxygen species
